# The Improved Health Outcomes Program (iHOP): A Unique Model to Promote Provider-Driven Research in a Medicaid Population

**DOI:** 10.3390/ijerph17145079

**Published:** 2020-07-14

**Authors:** Tara J. Schapmire, Jill Bell, Mark P. Pfeifer

**Affiliations:** 1 School of Medicine, University of Louisville, Louisville, KY 40202, USA; mark.pfeifer@louisville.edu; 2 Passport Health, 5100 Commerce Crossings Dr, Louisville, KY 40229, USA; Jill.Bell@passporthealthplan.com

**Keywords:** community-based research, Medicaid, community grants, academic partnerships, insurer and academic partnerships, community health, population health, public health promotion, child and/or maternal health, chronic conditions, mental health, preventive health, screening, system effectiveness, refugee health, Latino health, rural health, substance use disorders

## Abstract

We describe an effort to improve the care of Medicaid and uninsured individuals through a three-way partnership between a Medicaid managed care insurer, front-line providers, and an academic university. The project provided annual funding over eleven years, for research, pilot programs, and demonstration projects. Projects were provider-driven in design and methods. The Medicaid-managed care insurer-funded proposals were vetted by a neutral university team experienced in grant writing and community-based research and scored by a community-based review panel. The grant program ran from 2007 to 2018, funding 41 projects, totaling USD 2,097,842. The partnership of an insurer, a university, and frontline providers was not only viable and sustainable for over a decade, but also flexible, free of project selection issues, and well-received by all stakeholders. Funded providers worked in both urban and rural settings and included hospitals, community non-profits, outpatient clinics, academic and community health partnerships, and public health agencies. The projects generally reflected common issues in the Medicaid and uninsured population needs, such as childhood obesity, and they were consistent with the targeted goals of the program. Broad health foci included child and/or maternal health, chronic conditions, mental health, preventive health, screening, system effectiveness, special populations including refugees, Latinos, and rural individuals, and substance use disorders. Details of the awarded grantee goals, the grants management process, and lessons learned from the partnership are presented. The partnership triad model was effective and stable, with each partner adding unique value. The use of the academic institution to administrate the program provided an arms-length relationship between the insurer and the providers in project selection and allowed assistance to less experienced researchers in community settings.

## 1. Introduction

Improving population health outcomes is a complex challenge requiring commitment and action by multiple stakeholders, including communities and community providers, health systems, academia, and government [1]. Academic institutions with health science programs can have particular influence in bringing stakeholders together around this challenge but cannot singularly address community health. Community providers best understand barriers and opportunities to improve care at the patient level, yet lack resources, experience, and partnerships to accomplish solutions on their own. Medicaid insurers, through payments for clinical care, support the health of specific populations who are at particular risk from the social determinants of health, including children, pregnant mothers, seriously ill, mentally ill, and disabled populations. Improving the models and methods of clinical care could provide these beneficiaries with improved health outcomes while making the Medicaid model more efficient and effective. Yet, a review of the academic literature failed to reflect examples of similar models designed to achieve these goals.

The complex interaction of social determinates of health, the burden of chronic diseases, unachieved preventive health interventions, population health behaviors, access to health care, special populations, and health disparities drives the need for such partnerships. For example, 7 out of every 10 deaths among Americans each year result from chronic diseases such as heart disease, cancer, and diabetes; these illnesses account for 75 percent of the nation’s health spending [2]. Yet many of the risk factors that contribute to the development of these diseases are preventable or can be better managed [2,3]. Health starts in peoples’ homes, schools, workplaces, neighborhoods, and communities, influenced by individuals’ ability to take care of themselves by eating well and staying active, not smoking, getting the recommended immunizations and screening tests, and seeing a doctor when they are sick. Health is also determined in part by access to social and economic opportunities; the resources and supports available in homes, neighborhoods, and communities; the quality of schooling; the safety of workplaces; the cleanliness of water, food, and air; and the nature of social interactions and relationships [4]. These conditions explain in part the disparity in health among different Americans and why, in general, Americans are not as healthy as they could be [4,5,6].

We brought three domains together for this project to effectively leverage frontline caregivers’ (including academic hospitals, community non-profits, outpatient clinics, academic and community health partnerships, and public health agencies) experience and knowledge—one vital part of population health—to improve the health of Medicaid beneficiaries. In this paper, we describe the design, implementation, and process-related results of this eleven-year, state-wide program sustained by a Medicaid insurer partnering with frontline providers and an academic institution as a neutral intermediary. Providers proposed, developed, and tested alternative models of care delivery—many of which addressed social determinants of health—and generated better understanding of the unique needs of their patients. The feasibility, effectiveness, and lessons learned from such a program are described.

## 2. Materials and Methods

### 2.1. The Partnership

**Figure 1:**
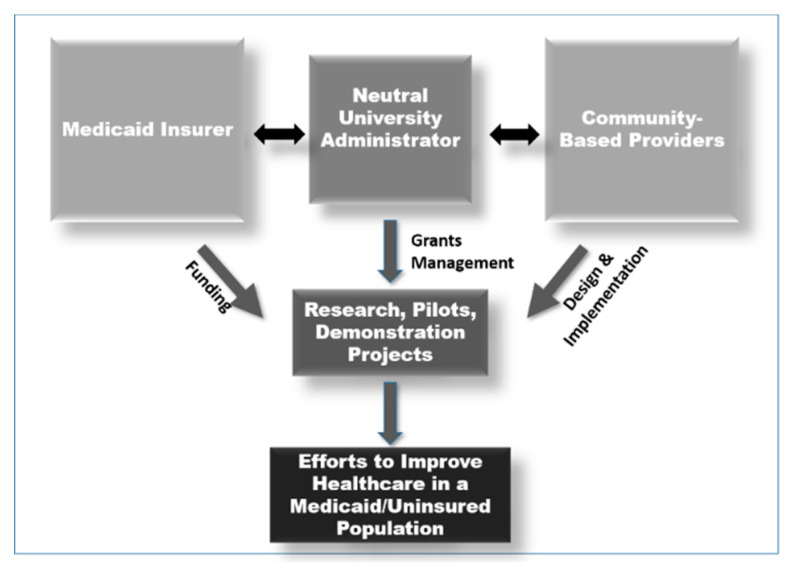
The Improved Health Outcomes Project (iHOP) model.

With a motivating goal to improve the health of their constituents, Passport Health Plan, a Medicaid-only insurer in Kentucky, contracted with the University of Louisville in 2006 for the leadership and administration of the jointly designed Improved Health Outcomes Project, iHOP. Figure 1 represents the partnership model. The projects would focus on the Medicaid and uninsured populations within the Commonwealth of Kentucky. Passport Health Plan provided an annual budget for the program.

The university was selected to administer the program for several reasons. Academic institutions have deep experience in research design and methodologies as well as expertise in proposal review and selection. In addition, a public university is a “neutral” third party that provides an arm’s length relationship between the funding source (the insurer) and the awardees (the clinical providers) outside of their existing payor–payee relationship. Any concerns about potential influence and favoritism were removed at the outset.

The community was engaged by annual announcements for projects using all possible means, including the insurer’s partnership council of representative clinical providers, email blasts, direct contact with large Medicaid provider groups, newsletters, and community meetings.

The iHOP program ended in 2019 when Passport reorganized its community outreach into a foundation model to provide more comprehensive community engagement and support.

### 2.2. The Process

The Improved Health Outcomes (iHOP) [7] program began in 2007 with the explicitly stated goal to improve the care of Medicaid and uninsured patients through research, pilot programs, and demonstration projects initiated by front-line clinicians involved in their care. The specific aims of the program were to support projects that: (1) lead to a better understanding of the needs, access issues, and quality of care in this population; (2) design and test models for or programs to improve the quality of care, satisfaction with care, access to care, and cost effectiveness and efficiencies of providing care; (3) explore and test unique collaborations and partnerships that offer opportunities to improve the care for this population including community-based initiatives; (4) design and test care management programs; and (5) design and test programs to address health care disparities.

The catchment area for this work mirrored that of Passport Health Plan, the sole-source Medicaid insurer based in Louisville, Kentucky, initially serving 13 surrounding counties from 1997–2013. Thus, iHOP projects for the first seven program years originated from this region where Passport served approximately 150,000 beneficiaries. In 2014, with changes in Kentucky’s Medicaid structure, Passport expanded to a statewide and non-sole source program, and the iHOP program likewise expanded its catchment area. In 2018, Passport enrolled approximately 320,000 of Kentucky’s 1.2 million Medicaid recipients (27%).

Each annual cycle began with announcing the funding cycle and application instructions through methods described above. A website supported any potential applicants with key dates for the process, frequently asked questions, and previously funded projects as a reference. In addition, the university provided guidance to applicants, most often sought by frontline clinical groups without a significant background in grant applications.

There were two phases of application review and selection. First, a letter of intent methodology was used to allow simpler entry into the process, encouraging a larger number of applicants to test their ideas. Letters of intent were restricted to two pages and stated the key objectives, methods, and outcomes evaluation for the proposed project. From that pool of letters of intent, which numbered 28–51 over the life of the program, 6–16 proposals were selected for full application, and up to five were funded annually.

Both the letter of intent and full submission review processes followed the same protocol, again derived from standard National Institute of Health (NIH) methods [8]. Committee members scored the applications individually, blinded to other members scoring until the face-to-face review committee meeting. At that time, scores were discussed, comments and questions were exchanged, and individual members could alter their scores based on these discussions. This led to a rank-order list of all projects. The number of applications recommended for full submissions, and later for funding, depended on the amount of annual funding available from Passport for the given year. A general guideline of being able to fund at least one in four full submissions was used. Feedback from the review committee proceedings could also be provided to unsuccessful applicants to improve future submissions.

The review committee evaluating applications and recommending funding consisted of clinicians, non-profit leaders, and public health leaders and broadly represented the region. All members were volunteers. This committee was jointly appointed each year by Passport Health Care and the university.

A conflict of interest protocol was again modeled after NIH processes [9]. No individual on an application in any role could serve on the review committee for that cycle. Individuals from organizations that had applicants were excused from any part of the review for applications from their organization.

Consistent with the stated goals, projects were selected that involved improving the care of both Medicaid and uninsured patients, given the often similar environments for care and the co-mingled populations. Projects were provider-driven in design, and no limitations or suggestions were made in either content methods beyond the above-stated broad aims. Funded projects required a progress report at six months, to be judged for satisfactory progress by the university project managers, before the second half of the funded amount was released. A final progress report was due two months after project completion.

A review of the program took place annually after each funding cycle. While few changes were required over the eleven years, minor modifications in the amounts of individual and total awards were adjusted periodically, and at least one award was required to address health issues of rural constituents beginning with the third cycle.

## 3. Results

The iHOP program ran ten annual cycles from 2007 to 2018. During this time, 41 one-year projects were funded, totaling USD 2,097,842. Initially, projects were capped at budgets of USD 50,000, then increased to USD 75,000 in 2016. From 2017 to 2018, project budgets were not capped but recommended to stay below USD 50,000. Projects targeting rural populations represented 20% of the awards. For the ten years of the iHOP program, projects ranged from USD 29,200 to USD 75,000 in funding. No-cost extensions (NCEs) were required for 29 of 41 (70%) of the projects, allowing projects more time to complete their work without additional funding. Taking NCEs into account, project durations ranged from one to three years, while most were completed in under two years. The iHOP program was intentionally not proscriptive regarding specific projects, wanting front line organizations to generate the projects based on their experiences and populations’ needs.

Table 1 highlights the funded projects by cycle and lists the types of organizations funded, broad health focus, target populations of projects, and project goals. Organizations awarded included academic hospitals (3/41; 7.3%), community non-profits (10/41; 24.3%), academic outpatient clinics (12/41; 29.2%), community out-patient clinics (2/41; 4.9%), academic and public health partnerships (2/41; 4.9%), academic and community health partnerships (10/41; 24.3%), an academic and governmental partnership (1/41; 2.4%), and a rural public health agency (1/41; 2.4%). Broad health foci included child and/or maternal health (19/41; 46.3%), chronic conditions (11/41; 26.8%), mental health (9/41; 21.9%), preventive health (7/41; 17.0%), screening (1/41; 2.4%), system effectiveness (6/41; 14.6%), special populations including refugees, Latinos, and rural individuals (9/41; 21.9%), and substance use disorders (2/41; 4.8%): note that many fit into more than one health focus.

The projects generally reflected common issues in the Medicaid and uninsured population needs, such as childhood obesity, and they were consistent with the targeted goals of the program.

## 4. Discussion

We have described a partnership to improve a Medicaid population’s health care through a community grants program. Projects most often awarded were partnerships between academics and community, public, and government health agencies, followed by academic outpatient clinics. This was likely because this university is a primary provider to the Medicaid, under-, and uninsured individuals in this urban environment surrounded by rural counties. Another explanation could be the university’s strong history of partnerships with the local community and surrounding counties to address social determinants of health. We were pleased with the wide variety of health foci as well, with roughly half focusing on child and maternal health while the other half focused on other age groups with varying health challenges. Each project focused on different aspects of social determinants of health, from access and education, to community engagement and food security. Many valuable lessons were learned, and we highlight them below for providers, Medicaid insurers, and for academic universities.

### 4.1. Lessons for Providers

Frontline providers and organizations can design, develop, and implement research pilots to improve the care of their patients. Community interest in this program was strong and sustained for more than a decade. Assistance to guide, encourage, and focus some of this frontline enthusiasm was important, and in our partnership was provided by the university. In addition, busy providers need easy “access” into these programs and the letter of intent process allowed that with minimal initial effort. With a broad variety of clinical organizations applying, some with minimal or no experience in “research” methods, and some very modestly sized, assistance and support were critical to design effective and doable projects. The care of Medicaid and uninsured patients in academic settings, such as primary care, is frequently not in a research-rich environment, often lacking both research experience and infrastructure. Thus, even academic clinical providers can benefit from the support provided in this model to develop and evaluate new approaches. This concept of working with applicants, as opposed to simply judging their submissions, is critical for community-based initiatives. Many projects carried out in this program allowed clinical organizations to test clinical approaches in their unique settings before further modification and implementation. Non-academic providers can partner successfully with academic researchers to design, implement, and test their ideas for improving care. Finally, learning what does not work is an equally important outcome for providers.

### 4.2. Lessons for Medicaid Insurers

Informed by both their experienced operations and extensive knowledge of the Medicaid services environment, the insurer provides key high-level guidance to these programs. At the same time, our third-party administration model offered the insurer many advantages. Medicaid organizations are political organizations by their very reliance on state funding; the model maintained neutrality and prevented influence and favoritism in project selection and funding. The third-party model not only spared the insurer day-to-day management in a realm not typically within their scope of work, but also provided rigor in processes and confidence in the—at times—difficult funding choices. This included conflict of interest and blind review processes derived from NIH models. Finally, the insurer’s community engagement was evident and measurable in this project.

### 4.3. Lessons for Academic Universities

Initially, numerous and long hardcopy applications produced extensive administrative work for the review process that distracted from other important support goals for the program. Thus, after two years, both letters of intent and full applications had pages limits and required sections. In addition, a paperless submission process streamlined the administrative burden. A dedicated website is an important tool for these programs. Clearly highlighting program goals while providing example projects, instructions, and frequently asked questions proved very useful for both applicants and administration. Finally, it is incumbent on the university serving in the grants management role to help ensure the funded projects adequately represent the population served; in our case, this was a mix of urban and rural individuals.

Some general lessons were also learned. Clinical organizations underestimated the impact of institutional review board processes, staff changes, and enrollment issues. A few projects were cancelled, primarily due to staff turnover and unexpected competing priorities, and are likely a normal part of community-based research. The two-phase funding and progress report method was successful in keeping projects on track. An all-volunteer review committee was feasible, engaged, and effective. Finally, grant periods of at least two years are recommended to complete community-based projects.

## 5. Conclusions

We did not attempt, in the scope of this project, to report the results of direct community impact, but rather have described an effort to improve the care of Medicaid and uninsured individuals through a partnership between a Medicaid managed care provider and an academic university to provide funding for research, pilot programs, and demonstration projects initiated by providers involved in their care. We found such a partnership leveraged the strength of each partner to create a feasible, sustainable, widely accepted, and very effective program to generate and carry out provider-initiated, community-based projects.

## Figures and Tables

**Table 1:** Characteristics of funded projects.

Cycle	Organization Awarded	Broad Focus	Target Population	Goal
I	Academic hospital	Child Health Maternal Health	New mothers and infants	To determine the impact of patient education aimed at increasing the number of new moms choosing to breastfeed
	Community non-profit	Special Populations	Low-income and uninsured refugees and immigrants	Health assessment of health care needs and experiences with the existing health care system, and determine barriers to health care access for this population, including both personal and environmental challenges faced when attempting to obtain health care and achieve wellness
	Academic outpatient clinic	Maternal Health Mental Health	Post-partum women 13–18 years of age with symptoms of depression	Study is to determine the feasibility, acceptability, and efficacy of the New Mother Program (telephone-based depression care management)
	Academic outpatient clinic	Child Conditions	High-risk children, 12–27 months	To improve access to preventive dental care in the first two years of life and to reduce the risk of early childhood caries
	Academic outpatient clinic	Chronic Conditions	Low-income, uninsured/underinsured individuals with chronic illness	To improve clinical outcomes for individuals with chronic illnesses, with a focus on diabetes, through the implementation of an on-site chronic care coordinator
II	Academic hospital and public health partnership	Screening/Prevention	Oral health practitioners	Feasibility study to develop and test an educational intervention aimed at oral health practitioners’ ability to improve recognition of early-stage oropharyngeal cancers in African Americans
	Academic outpatient clinic	Chronic Conditions	Adult African Americans with type 2 diabetes mellitus	To determine the effects of a motivational interviewing intervention on adherence to prescribing treatment regimens, diabetes markers, and number of unscheduled health care visits
	Rural outpatient pediatric practices	Preventive Health	Obese children on managed Medicaid care plan and their committed parent	To determine the effects of an interdisciplinary educational intervention on healthy behaviors related to diet and exercise
	Academic and community partnership	System Effectiveness	Persons calling 911 for problems triaged as non-life threatening	To implement and measure the effects of a pilot program providing alternative medical transport care to patients who access the 911 system for non-life-threatening illnesses or injuries
	Academic hospital	Maternal Health	New mothers presenting in labor who request intrauterine devices placement post-partum	A randomized, controlled trial comparing typical placement of intrauterine devices at six weeks post-partum or later vs. immediate post-placental placement on the rate of success, complications, and patient satisfaction
III	Partnership of academic hospital with several rural community hospitals	Child Health Maternal Health	Maternal healthcare providers and new mothers and infants	To increase breastfeeding rates in sixteen counties by training healthcare providers to implement a patient education program at the ten birthing hospitals served by a managed Medicaid care plan
	Academic outpatient clinic	Maternal Health Mental Health	Teen mothers with depression	To determine the acceptability, feasibility, and efficacy of a public health, social marketing intervention to improve health care use of teen mothers with symptoms of depression
	Academic and community partnership	Child Health Maternal Health	Low-income pregnant women	Feasibility study of a prenatal mothers’ day out program
	Community non-profit	Chronic Conditions	Low-income/uninsured individuals	Feasibility of a pilot indigent prescription program
	Academic and public health partnership	Special Populations	Latino community	To develop, implement, and evaluate a community-focused, digital storytelling tool, aimed at engaging community members in the elimination of health inequities
IV	Academic hospital	System Effectiveness Chronic Conditions	Medicaid and uninsured individuals	To understand the extent to which guardianship services achieve cost savings and improved access to health care
	Academic outpatient clinic	Mental Health	Medicaid patients with major depression seen in primary care clinics	Pilot study to improve the diagnosis and treatment of major depression in primary care settings
	Academic and community partnership	System Effectiveness Chronic Conditions	Medicaid and uninsured individuals	To improve the health outcomes and reduce readmissions through a community care navigator-led disease management initiative
	Academic outpatient clinic	Child Health	Children with asthma and their caregivers	Feasibility study of asthma education program on knowledge, behavior, and quality of life
	Academic outpatient clinic	Maternal Health Substance Use Disorders	Pregnant opioid users	Pilot feasibility study on program aimed to improve opioid treatment and outcomes for pregnant women and their babies
V	Academic and community partnership	Child Health Maternal Health	At-risk parents and children	Pilot feasibility study of the impact of a parent training intervention on parent/child emotional, behavioral, and developmental outcomes
	Academic outpatient clinic	Preventive Health	Smokers who visit family medicine clinics	To compare the effects of resident physician motivational interviewing (MI), resident physician MI plus registered nurse (RN), and the standard of care counseling approach on current smokers’ smoking behaviors (readiness to quit, cigarettes smoked per day, current smoking rates), self-efficacy to quit smoking, and nicotine dependence
	Academic and community partnership	Special Populations Chronic Conditions	Rural Latinos with diabetes	Pilot feasibility study of the impact of a comprehensive, peer navigator-led diabetes education program combined with a food pantry on attitudes, behaviors, and clinical diabetic outcomes
	Community non-profit	Special Populations	Refugees	Exploratory study of refugee health needs, perceived quality of life, and barriers to accessing healthcare aimed at developing a community health worker program
	Community non-profit	Preventive Health	Medicaid/uninsured residents served by a housing assistance program	Pilot study of a community engagement program aimed at addressing prevention education on diabetes and heart disease
VI	Rural public health agency	Child Health Preventive Health	Elementary school students	Pilot study of the impact of an after-school nutrition and recreation educational program on knowledge and behaviors
	Academic and community non-profit partnership	Mental Health	Adults with severe/persistent mental illness	Develop and implement a tracking and reporting system to assess the impact of supportive housing for adults with severe/persistent mental illness on the traditional health care and criminal justice systems, as well as utilization of other social services, and overall client life satisfaction
	Multiple rural community non-profits	Maternal Health Mental Health Substance Use Disorders	Pregnant mothers with mental illness, domestic violence, substance abuse/dependency issues in primary care settings	Develop and implement a peer support specialist program aimed at bridging mental health services with primary care services for at-risk pregnant moms
	Rural outpatient pediatric practice	Child Health Preventive Health	Overweight/obese children aged 4–15	Study aimed to improve the overall health and quality of life of children via an intervention involving regular exercise, dietary changes, mental health counseling, and family education
VII	Rural hospital	Chronic Conditions System Effectiveness	Hospital patients at high risk for 30-day re-admission	Pilot study of the impact of a lay health worker intervention on hospital readmission rates
	Academic and community partnership	Special Populations Preventive Health	Rural Latinos with diabetes	Develop a culturally sensitive preventative medical home without walls in a Latino food pantry that includes a lay health worker
	Academic and government partnership	Child Health Mental Health	Children on Medicaid with mental and/or behavioral health issues	Exploratory study aimed to describe prescribing practices for antipsychotic medications (APM) for children < 5 years of age and for children receiving multiple, concurrent APM including age of the children, provider type, adherence to recommended lab testing, and use of behavioral health interventions
	Rural community non-profit	Chronic Conditions System Effectiveness	Rural clinic patients on Medicaid or uninsured	Test the impact of an intervention designed to proactively identify and close gaps in preventive care provision, including cancer screening utilization and chronic disease management, through the strategic use of information technology (e.g., electronic health records), the development of new workflows and staff training, and continuous quality improvement
VIII	Community non-profit	Special Populations Chronic Conditions System Effectiveness	Refugees	Design and test a chronic disease self-management project aimed at improving healthcare access and health outcomes through a community health worker model
	Academic outpatient clinic	Child Health Maternal Health Mental Health	Children on Medicaid with special needs and their female caregivers	Test the effects of brief cognitive behavior therapy (CBT) for low-income female caregivers with depressive symptoms who have a Medicaid enrolled child with special health care needs in improving child and caregiver outcomes
	Academic outpatient clinic	Child Health	Low-income infant–mother dyads	Pilot test of a community-based intervention to increase breastfeeding initiation, duration, and exclusivity
	Rural community non-profit	Child Health Preventive Health	Pediatric clinic patients	Implementation of the proactive office encounter model aimed at improving preventative health measures and efficiency of care
IX	Academic outpatient clinic	Child Health Mental Health	Infants 0–24 months	Exploratory project to detect children at risk for and suffering from toxic stress and provide interventions that strengthen families and promote resilience
	Community non-profit	Chronic Conditions System Effectiveness	Managed Medicaid recipients at high risk for hospital readmissions	To discover whether home visit interventions made by a community paramedic could improve health outcomes and also lower overall healthcare spending
X	Academic and community partnership	Special Populations Chronic Conditions Mental Health	Refugees with a known history of at least one chronic health condition and one serious mental health issue and their identified/designated family health broker	To test the benefits of utilizing family health brokers (a safe and trusted intermediary family member functioning as a go between one’s family and health care providers) as part of treatment to improve the overall health/mental health of refugees with chronic disease conditions and serious mental health issues
	Academic and multiple rural hospitals partnership	Child Health Special Populations	Parents of rural, remote, and underserved children with communication disorders on Medicaid	Pilot test a telehealth project aimed at increasing access to high-quality clinical speech language pathology services and leveraging parent support
